# Municipal strategies and meeting minutes’ descriptions of the promotion of children’s mental health: a document analysis

**DOI:** 10.1177/1403494820961902

**Published:** 2020-10-08

**Authors:** Outi Savolainen, Hannele Turunen, Marjorita Sormunen

**Affiliations:** 1 Department of Nursing Science, University of Eastern Finland, Finland; 2 Institute of Public Health and Clinical Nutrition, University of Eastern Finland, Finland

**Keywords:** Mental health promotion, children, municipality, decision-making, document analysis

## Abstract

**Aims:**

Little is known about how municipal strategies, programmes and plans pay attention to the promotion of children’s mental health and whether it is discussed and reported in the municipal councils, boards and committees. The purpose of this study was to examine how municipalities in one Finnish region promote mental health, with a focus on the promotion of children’s mental health.

**Methods:**

Document analysis was used as a research method. Documents were selected for a one-year period (2018) from three municipalities of the North Savo region. Analysed documents (*n*=269) were municipal strategies, programmes and plans, as well as meeting minutes of municipal councils, boards and committees. Eight domains of the structural indicators of mental health were used as an analysis frame.

**Results:**

In total, 1169 mentions related to the structural indicators of mental health were found in the documents. In strategies, programmes and plans, parenting-related mentions were found most often. Regarding the minutes, the issues discussed and reported about the wellbeing of children focused on practical issues, such as the construction of day care buildings.

**Conclusions:**

**Document analysis indicated that mental health promotion involved mostly the society and environment and not as much the age and setting. There was a lack of mentions regarding preschool experiences and family support/childcare and the promotion of mental health through schools and education, especially in the meeting minutes of municipal councils, boards and committees.**

## Introduction

The basis of mental health (MH) is built in childhood and adolescence. At best, the growing environment supports MH and provides an opportunity for positive development of mental resources. However, children’s and young people’s starting points for growth and development can be variable due to social inequalities and the accumulation and transmission of problems to the next generation. The importance of the childhood phase for the wellbeing and health of people throughout their lives needs to be recognised to support effectively the MH of children and adolescents in their daily lives [1].

MH is ‘a state of well-being in which the individual realizes his or her own abilities, can cope with the normal stresses of life, can work productively and fruitfully, and is able to make a contribution to his or her community’ [2]. This description emphasises the positive aspects of MH, which will also be the focus of this paper. Negative MH (or mental ill-health) is another aspect, and it encompasses a continuum from the most severe disorders to a variety of common MH problems [3]. Determinants of MH, in turn, are factors that influence MH either positively or negatively [4]. Although an individual can influence MH with their lifestyle choices, such as a healthy diet and adequate sleep, society still has a major impact on the population’s promotion of MH [5–7]. The socio-ecological model of health promotion [8], as the theoretical framework of this study, assumes that an individual is an integral part of the larger sociocultural, economic and political environment and health behaviour develops in interaction with the socio-ecological environment. Therefore, it is important to investigate how this is considered at the public policy level.

The goal of MH promotion is to strengthen mental wellbeing, resources and resilience, and influence the social determinants of MH by creating supportive living conditions and environments and reducing factors harmful in terms of MH. Social inequality plays a key role in society’s efforts to promote children’s wellbeing. However, there are major differences between societies in the types of support available and the extent to which support is provided. In the Nordic countries, income transfers and services designed to reconcile work and family reduce the risk of poverty [9]. Even so, in Finland, 10% of families with children live below the poverty line [10] and, therefore, are in vulnerable positions regarding MH.

Childhood experiences before school age are significant to an individual’s later development and MH. Essential in this context are the factors associated with the home and the parents [4], such as maternal depression during pregnancy [11, 12], birth to young parents [13, 14], poverty, unsatisfactory social relationships [14–16], loneliness [17] and maltreatment [18]. Thus, early childhood is the most favourable for effective MH-promoting activities. School is another context in which MH activities can be well integrated into daily life. Hence, school life has a great potential in MH promotion [19].

It is the responsibility of the state and municipalities to ensure inhabitants live in an environment that maintains and protects their MH. With regard to children, this is required by the United Nations Convention on the Rights of the Child [20], which obliges us to consider the best interests, rights and views of the child. In Finland, municipalities produce general welfare programmes as well as plans targeting different population groups and cross-governmental themes [21]. Strategies, programmes and plans are mainly prepared by the authorities and approved by the municipal council. The statutory municipal strategy is the most important guidance tool in managing the municipality. Welfare reports, in turn, gather information from various sources on the health and wellbeing of residents and related factors. These documents, among others, guide the activities of the municipality [22]. However, there is a gap in knowledge about how municipalities’ strategies, programmes and plans pay attention to and enhance determinants of MH related to the society, environment and individual.

The structure of municipal decision-making organs is based on the Local Government Act of Finland [22]. Depending on the size of the municipality, there are from 13 to more than 79 representatives in the municipal councils. Councils appoint members of the municipal boards, the number of which is determined based on the municipality’s statutes. Councils also set up the boards that carry out tasks in their fields (for instance, social and healthcare, sports, culture and education). The boards’ names and areas of responsibility might be different in different municipalities. Municipal councils, boards and committees deal with issues, many of which affect children’s wellbeing, either directly or indirectly. Municipal planning and decision-making needs to consider age-specific needs so that the developmental tasks associated with each age can be supported as well as possible [23]. Consequently, knowledge about the issues discussed and reported in the municipal councils, boards and committees in this context is important. The purpose of this study was to examine how municipalities in the region of North Savo promote MH, with a focus on the promotion of children’s MH. Furthermore, the purpose was to answer the following research questions:

- How the promotion of children’s MH is visible in the strategic documents of municipalities?
- How the promotion of children’s MH is documented at various levels of municipal decision-making?

## Methods

### Case description

The region of North Savo is located in eastern Finland, and it is about 20,367 km² in size. It has a population of 245,602 inhabitants, with a density of 14.6 inhabitants per km², and it is the sixth largest region in Finland. North Savo has 18 municipalities, each with a population of 1500–119,000 [24]. In this region, mental disorders are more common than in the rest of Finland. In addition, the region has the highest morbidity index compared to the morbidity of the population of the Finnish regions at the national level [25].

### Materials

The data collected for this document analysis included: (a) strategies, programmes and plans of the municipalities; and (b) all minutes of municipal councils, boards and committees for a one-year period (2018), except minutes of municipal audit committees and central municipal election boards (Table I).

**Table I.** Selected documents.

Document	No. of documents
Strategies, programmes and plans	23
Regional welfare report of North Savo 2018–2021	1
Strategy of the municipality	3
Local welfare report	3
Plan for mental health and substance abuse work	3
Municipal plan for the wellbeing of children and young people	3
Operating and financial plan	3
Municipality-specific early learning plan	3
Municipality-specific curriculum	3
Integration programme	1
Minutes	246
City/municipal councils	20
City/municipal boards	71
Boards and committees	155
Total	269

Documents were selected from three municipalities of the region: one with a large population (>100,000 inhabitants), one with a medium population (10,000–100,000 inhabitants) and one with a small population (<10,000 inhabitants). Selection was done to estimate the relevance and reliability of the documents [26]. The total number of documents was 269: 126, 63 and 79 documents from municipalities A, B and C, respectively. In addition, the statutory regional welfare report of the region was selected. The documents were freely available from public web pages.

### Analysis

Qualitative document analysis was used because it allows systematic and retrospective review of existing data [27]. In addition, descriptive statistics (percentages and frequencies) were used [28].

All eight domains of the structural indicators of MH from an earlier MINDFUL [4] project (MH information and determinants for the European level) were included to obtain an overall picture of MH promotion. The analysis frame included 53 recommendations to improve the conditions for MH (Table II). This paper focuses specifically on mentions related to two domains of children’s MH promotion: preschool experiences and family support/childcare, and promotion of MH through schools and education. These two domains were selected because they relate directly to the daily life of children.

**Table II.** The eight domains of the structural indicators of mental health,^a^ and related mentions from documents of three municipalities (*f*=1169).

The domains of the structural indicators of mental health (*f* and % of all mentions)	Recommendations to improve the conditions for mental health (*f*)	Mentions (*f*)	
		Strategies, programmes and plans	Minutes
Preschool experiences and family support/childcare (96; 8.2)	Comprehensive motherhood care (5)	3	2
	Parenting education (15)	14	1
	Paid parenthood leave (0)	0	0
	Comprehensive postnatal care (2)	2	0
	Day care for children (32)	9	23
	Support services for parents at risk (42)	34	8
	Total	62	34
Promotion of mental health through schools and education (90; 7.7)	Integrating mental health promotion and mental health issues into the school policy and curriculum (1)	1	0
	Providing psychological support for pupils (20)	20	0
	Providing support for teachers (6)	6	0
	Involving parents (32)	32	0
	Fostering teamwork (30)	29	1
	Implementing health promoting school programmes (1)	1	0
	Total	89	1
National mental health framework (117; 10.0)	Modern mental health legislation (0)	0	0
	Mental health policy analysis (0)	0	0
	Mental health programme (10)	6	0
	Cooperation between different sectors (66)	64	2
	Active human resource policy (19)	16	3
	Inclusion of users and caregivers (20)	20	0
	A comprehensive mental health information system (0)	0	0
	Anti-stigma programme (0)	0	0
	Mental health impact assessment (0)	0	0
	Research in mental health policy and promotion (0)	0	0
	Proper financing (2)	2	0
	Total	108	9
Employment and workplace mental health (91; 7.8)	Comprehensive employment policy (24)	15	9
	Enhancing communication and personnel involvement (4)	3	1
	Implementation of antidiscrimination provisions (3)	0	3
	Providing management skills training (4)	4	0
	Implementing workplace health promoting programmes (4)	1	3
	Adjusting between work and family life (4)	3	1
	Supporting the unemployed or those in precarious work situations (47)	35	12
	Providing supported employment for people with mental disorders (1)	0	1
	Involving the trade unions (0)	0	0
	Total	61	30
Social capital: mentally healthy communities (331; 28.3)	Enhancing participation (111)	95	16
	Supporting the establishment of self-help activities (19)	19	0
	Providing support systems (116)	85	31
	Access to mental health services (29)	23	6
	Enhancing equity and social justice (56)	35	21
	Total	257	74
Physical environment (139; 11.9)	Building mentally healthy housing environments (30)	12	18
	Establishing parks and other green spaces (17)	14	3
	Providing playgrounds for children (7)	2	5
	Reducing noise and crowdedness (11)	2	9
	Securing public safety (74)	70	4
	Total	100	39
Leisure activities (215; 18.4)	Free-time education (12)	8	4
	Sporting facilities (106)	52	54
	Culture (55)	40	15
	Facilities for civic participation (27)	24	3
	Youth organisations (12)	4	8
	Activity centers for children and families (3)	3	0
	Total	131	84
Mental health and older adults (90; 7.7)	Enhancing social participation (14)	10	4
	Preventing loneliness and social isolation (8)	6	2
	Providing opportunities for independent living (54)	42	12
	Providing appropriate health and social services (13)	7	6
	Combating ageism (1)	0	1
	Total	65	25
Grand total	1169	875	294

a In accordance with Lehtinen, 2008.^4^

In the analyses, the material was tabulated first by document type (Table I) and sorted into two categories following the research questions: (a) the strategies, programmes and plans (strategic planning); and (b) the minutes (documented decision-making). Subsequently, the contents of the documents were categorised deductively into the domains and recommendations using the classification framework. The analysis proceeded with one researcher reading the documents to get an overview of them. Then, mentions related to the recommendations to improve the conditions for MH were identified. In strategies, programmes and plans, a mention was usually a single sentence or a paragraph a few sentences long. In the minutes, it was the subject of the present case. The text in the present case was read, and a decision was made on whether to pick up the mention. The data and analysis were reviewed regularly and frequently with the research team. Afterwards, the mentions were checked, and possible corrections were made to ensure consistent classification. Finally, statistical methods were used to describe the number of the structural indicators of MH-related mentions in the documents. An example of the mention classifications is described in Table III.

**Table III.** An example of the classification of mentions.^a^

The domain of the structural indicators of mental health	Recommendation to improve the conditions for mental health	Examples of the documents/topic and mentions	Document type	
			Strategies, programmes and plans	Minutes
Preschool experiences and family support/childcare	Day care for children	Plan for mental health and substance abuse work/objectives and proposals for action: the social service guides children to day care as a child support measure	x	
		Municipality-specific early learning plan/introduction: in the future, special attention will be paid to the early childhood education so that caregivers can evaluate the start of childcare	x	
		Integration program/action: depending on the needs of the family and the child, family day care and kindergarten services as well as open early childhood services are available	x	
		Minute of municipal council/council motion: inspection of municipal public and private early childhood education and care		x
		Minute of municipal board/issue discussed: position of the head of day care centre		x
		Minute of municipal committee/issue discussed: customer fees		x
	Support services for parents at risk	Regional welfare report of North Savo 2018–2021/focus areas, objectives and actions: supporting families with substance abuse problems	x	
		Municipal plan for the wellbeing of children and young people/description of services: home care for families with children is a short-term support and assistance in situations where the family is facing an acute life crisis or otherwise needs support in different situations of everyday life	x	
		Plan for mental health and substance abuse work/action: the maternity clinics and child healthcare put screening for domestic violence to use	x	
		Minute of municipal council/admitted proposal for a decision: social credit		x
		Minute of municipal board/issue discussed: establishment of new positions in the service area		x
		Minute of municipal committee/issue discussed: the reform of the family support centre		x

a In accordance with Lehtinen, 2008.^4^

## Results

### General characteristics of the municipal documents

**Figure 1.**
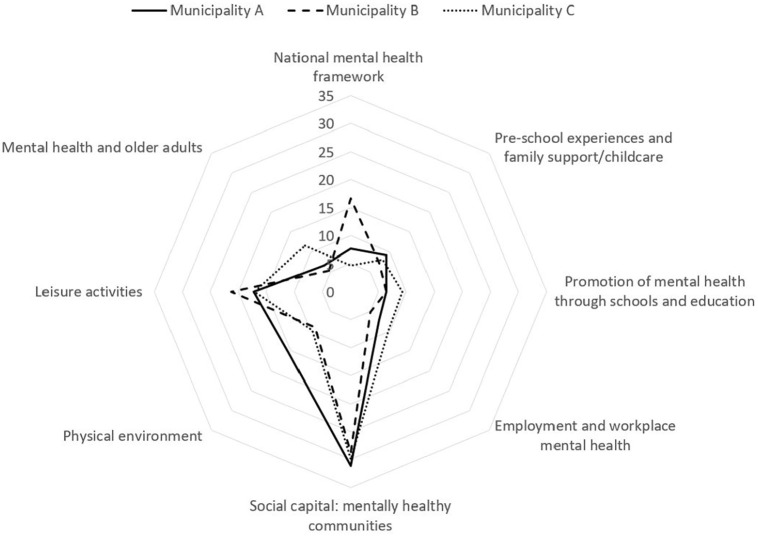
Mentions related to the structural indicators of mental health.

In total, 1169 mentions related to the eight domains of the structural indicators of MH were found from all selected documents of three municipalities (Table II). The highest number of mentions connected to the societal and environmental indicator social capital: mentally healthy communities (28.3%), followed by leisure activities (18.4%) and physical environment (11.9%). Whereas fewer mentions were reported about the age and setting-related indicators, such as the promotion of MH through schools and education (7.7%) and preschool experiences and family support/childcare (8.2%). Most mentions were found from the documents of the largest municipality. In addition, indicators were emphasised differently in each municipality. For example, municipality C, with a large elderly population, had the most mentions related to the MH and older adults domain. The radial diagram (Figure 1) presents the distribution of mentions into eight domains.

Strategies, programmes and plans included 875 mentions. In these documents, MH promotion was largely focused on the societal and environmental indicator social capital: mentally healthy communities (22.0%), and the mentions were related to the recommendations enhancing participation or providing support systems. Strategies, programmes and plans had the fewest mentions related to the age and setting-related indicators employment and workplace MH (5.2%), preschool experiences and family support/childcare (5.3%) and MH and older adults (5.6%).

Minutes included 294 mentions. The societal and environmental indicators also occurred most frequently in the minutes: leisure activities (7.2%), social capital: mentally healthy communities (6.3%) and physical environment (3.3%). Sporting facilities, support systems and mentally healthy housing environments were particularly emphasised under these indicators. As with the planning documents, there were fewer mentions related to the age and setting-related indicators, such as promotion of MH through schools and education (0.1%). The indicators preschool experiences and family support/childcare and promotion of MH through schools and education are presented in more detail below.

### The promotion of children’s MH in the strategies, programmes and plans of the municipalities

#### Preschool experiences and family support/childcare

Most of the mentions under this indicator were related to the support services for parents at risk (*f*=33). General level mentions especially highlighted early identification of risks, early support for families with substance abuse or MH problems and preventive work instead of repairing work. Practical recommendations included screening for domestic violence, rehabilitation periods for mothers who use substances, more intensive follow-ups for families with special needs and the development of collaboration in family services and project activities. Some of the mentions were related to the whole family, and emphasised parenting and family counselling, a home service for families and improvement of the accessibility of family services.

Mentions related to parenting education (*f*=14) mainly stated that parents should be supported. Practical proposals for action emphasised peer support, guidance and parenting support groups. In addition, mentions related to day care for children (*f*=9) unilaterally urged that all children should have access to day care. Mentions were also addressed that responded to the growing demand for day care. There were a few mentions in the documents related to comprehensive motherhood care (*f*=3) and comprehensive postnatal care (*f*=2). Mentions briefly described the operational objectives of the child health centres and the maternity clinics and noted that the child health centres should promote children’s health. Instead, there were no mentions under the indicator paid parenthood leave.

#### Promotion of MH through schools and education

Most of the mentions under this indicator were related to the recommendation involving parents/guardians (*f*=32). Involvement meant effective home–school collaboration, in particular a parenting committee, parenting evenings, peer planning, rulemaking, bullying and violence prevention and mediation, opportunities to influence school meals, participation in school activities and curriculum commentary and evaluation. Information and communication technologies, such as social media and the web interface for the school administration programme, were advised for use in the interaction between school and home.

Several mentions were also related to fostering teamwork (*f*=29). According to the documents, purposeful, regular, interactive and systematic collaboration and teamwork is key to planning and implementing schoolwork. Collaborative approaches should be developed with pupils, parents and other authorities and actors to promote the wellbeing of children and young people. Teachers are also encouraged to collaborate with other teachers regionally and across school levels and to offer support to classroom teachers during the pupils’ enhanced support periods.

Providing psychological support for pupils was mentioned rather often (*f*=20) and mentions dealt with a three-tier support procedure in basic education, the contribution and job description of the school psychologist and MH and life management courses, which a psychologist could provide for pupils. Some mentions suggested that schools should have psychiatric nurses who would also provide support for students. Fewer were the mentions related to the recommendation of providing support for teachers (*f*=6). The strategies, programmes and plans included one mention regarding the recommendation of implementing health promoting school programmes, which was about the establishment of a coherent substance abuse model for schools, as well as a recommendation integrating MH promotion and MH issues into the school policy and curriculum. Regarding the latter, the mention was related to the lessons provided by a school social worker and psychologist dealing with MH promotion and mental balance.

### The promotion of children’s MH in the minutes on municipal councils, boards and committees

#### Preschool experiences and family support/childcare

Based on the minutes, the promotion of children’s MH under this indicator was focused mostly on day care for children (*f*=9) and for very practical arrangements, such as the planning and construction of day care centres, the recruitment of personnel, day care fees and the arrangement of the shift care. In addition, council motion about the subjective right for day care and group sizes was discussed. In addition, the indicator support services for parents at risk stood out with several mentions (*f*=8), dealing with the establishment of positions, social credit issues and child protection policies.

Furthermore, many fewer actions, if any, were taken concerning other recommendations. With respect to the recommendation of comprehensive motherhood care, two council motions were discussed (*f*=2): free pregnancy prevention for women under 25 years of age and carbon monoxide measurement in maternity clinics for pregnancy monitoring. One council motion was discussed under the recommendation parenting education, which suggested support for those considering parenting. No mentions related to the recommendations paid parenthood leave or comprehensive postnatal care were found in the minutes.

#### Promotion of MH through schools and education

Within this indicator, only one mention was found from all the minutes. The mention was under the recommendation fostering teamwork, and it addressed the collaboration in developing student guidance. Consequently, there were no actions related to other recommendations under this indicator.

## Discussion

The results of this study indicate that the majority of the documented mentions were linked to the society and environment, while fewer were related to the age and setting. Consideration of the MH promotion varied between municipalities, so that while the largest municipality was planning different recreational activities, the smallest municipality plans focused on ensuring everyday activities. The results reflect the location, structure and strategies of each municipality. The administrative structure in each of the three municipalities was similar, including a municipal council, municipal board and committees appointed by the municipal council. However, the number of members varied in each of the municipalities depending on the population size. The number of written documents they produced also varied: the largest municipality had the largest number of documents. Thus, this could be why most mentions were found from the documents of the largest municipality.

In this study, all structural indicators of positive MH from the MINDFUL [4] project were used as an analysis frame. The frame also included domains that were not directly related to children, such as MH and older adults, to obtain an overall picture of how the indicators were emphasised in the municipalities. However, this paper focuses specifically on the domains of preschool experiences and family support/childcare and promotion of MH through schools and education. Participation related to all age groups was emphasised in the documents. In Finland, the current programme of Prime Minister Marin’s 2019 government [29] has the strong objective of strengthening civic participation. Hence, municipal policies were in line with the programme. In contrast, fewer were focused on the age and setting-related indicators, such as preschool experiences and family support/childcare. The Finnish government is also committed to strengthening child health centres, maternity clinics and family support. Thus, children’s participation should be made more visible at the local level, which means investing in early childhood and education. As expected, children’s MH promotion was reflected much more in the strategic documents than in the minutes. In the minutes, the issues to be dealt with were broader and more community wide, than related to the individuals, and more detailed decisions related to individuals are mainly made at the authority level.

Strategies, programmes and plans indicated that, under the preschool experiences and family support/childcare and promotion of MH through schools and education, the support services for parents at risk, parenting education and involving parents/guardians were highlighted. However, comprehensive motherhood care or comprehensive postnatal care emerged only a few times. As previous studies have indicated, the family circumstances of childhood have a major impact on the later wellbeing of children [11, 13–15, 18]. The support of parenting and families protects the children’s healthy growth and development and may obviate the need for child protection. MH issues should also be invested more in schools, where support is essential to children’s wellbeing. Things learned and experienced at school have a decisive impact on a person’s wellbeing, even at an older age. However, the analysis indicated that MH issues related to school were less received.

The promotion of children’s MH related to preschool experiences and family support/childcare and promotion of MH through schools and education was marginally documented in municipal decision-making. This indicated that written plans do not guarantee that things will be visible in practice. For instance, despite the strategies, the municipality may still decide to make budget cuts from wellbeing, which is short-sighted because the cost of prevention shifts to repairing the harm. In Finland, the financial cuts made during the 1990s economic depression increased the need for children’s MH services [30]. There will be situations that also threaten society in the future. The current coronavirus pandemic has had a significant impact on the wellbeing of children and realisation of their rights. MH problems were already a public health issue internationally and nationally. As the situation regarding the prevalence of MH disorders is even worse in the North Savo region than in the rest of Finland, more attention should be paid to the protection of children’s wellbeing in decision-making in this region. Based on the results of this study, it is not possible to draw conclusions about the causes of the prevalence of MH disorders in the region. However, the structures should be as good as possible to withstand changing circumstances.

It is important from the beginning that MH promotion be addressed during strategic planning. However, that alone is not enough, nor does it mean that things will happen in practice. The minutes pointed out what kinds of decisions and plans are made by councils, boards and committees. They provide guidelines but do not talk about all the MH promotion that is done in the municipality; rather, they speak specifically about the issues that are dealt with in municipal policy. Based on the results of this study, it is not possible to draw conclusions on how the frequencies of mentions correspond to actual decisions and actualise in real-life settings.

The MH of children could be improved, for example, by reducing child poverty in the family [10, 14–16] and making MH a part of the culture of early childhood education and schools [10]. However, lack of mentions regarding recommendations integrating MH promotion and MH issues into the school policy and curriculum and implementing health-promoting school programmes indicates that these factors are not sufficiently promoted in the strategic planning and decision-making of municipalities. The importance of MH consideration outside the social and healthcare sector at all levels of the socio-ecological environment is clear. Well-targeted actions could also reduce the impact of social inequalities on physical and MH [1]. Although the parents have the primary responsibility for the care of the child, the state and municipalities must support the parents and make the respect for the child’s rights a priority [20].

### Strengths and limitations

The main strength of this study is that, to the best of the authors’ knowledge, it is one of the few studies to examine the promotion of children’s MH based on official municipal documents. In addition, the official documents of municipalities are relatively reliable because the objects are socially identifiable. The use of original sources, as well as a comprehensive selection of documents for a one-year period, improves the reliability of the analysis [27]. Furthermore, the meeting minutes of all councils, boards and committees of the three municipalities, with the exception of minutes of municipal audit committees and central municipal election boards, were analysed.

The number of the mentions is guiding, as the selection is interpretative to some extent. In addition, some of the mentions could have fit with many of the recommendations on the analysis frame. Double-checking of documents by two or more researchers would have increased reliability. However, data and analysis were reviewed regularly and frequently with the research team to maintain consistency. The annexes of the documents were not analysed, nor were the decisions of the authorities contained in the minutes, because there was no background information available besides the title. Inclusion of the annexes and the decisions of the authorities could have produced a more complete picture. In addition, qualitative analysis could have deepened the results.

## Conclusion

This study provides new information about the promotion of children’s MH in the strategic planning and decision-making of municipalities. Document analysis indicated that MH promotion was mostly involved with the societal and environmental indicators and less with the age and setting-related indicators. From strategies, programmes and plans, parenting-related mentions were found the most. In contrast, recommendations for comprehensive motherhood care and paid parenthood leave remained almost completely unmentioned. Regarding the minutes, the issues discussed and reported related to the wellbeing of children focused on very practical issues. Overall, the minutes included just a few mentions related to the indicators preschool experiences and family support/childcare and promotion of MH through schools and education. Further research should examine whether similar results are found in other regions or countries. Policy analysis is evidently needed to investigate the coherence between the outlines in the strategies, programmes and plans and municipal decision-making. In the context of the COVID-19 crisis, it would be important to study in the coming years the effects of the economic depression that began in spring 2020 on children’s wellbeing.
